# Dairy and Cardiovascular Disease: A Review of Recent Observational Research

**DOI:** 10.1007/s13668-014-0076-4

**Published:** 2014-03-15

**Authors:** Beth H. Rice

**Affiliations:** Dairy Research Institute, 10255 West Higgins Road, Suite 900, Rosemont, IL 60018-5616 USA

**Keywords:** Dairy, Milk, Cheese, Yogurt, Cardiovascular disease, Coronary heart disease, Cultured dairy, Heart disease, Atherosclerosis, Stroke

## Abstract

The consumption of dairy, including milk, cheese and yogurt, has been associated with better quality of diet and reduced risk of cardiovascular disease, the leading cause of death globally. The purpose of this review is to examine recent literature on the relationship between dairy consumption and risk of cardiovascular disease incidence and mortality. Eighteen observational studies were reviewed, the results of which indicate that total dairy intake does not contribute to cardiovascular disease incidence or death. Based on available data, it appears that milk, cheese, and yogurt are inversely associated with cardiovascular disease risk. Data pertaining to dairy fat were inconclusive, but point to a potential protective effect of full-fat milk, cheese, and yogurt on risk of cardiovascular disease. Currently, there is a need to study specific well-defined foods, as opposed to calculating nutrients, in order to better understand these relationships. Future research need not replicate the body of literature on total dairy consumption and associated risk of disease, but rather should focus on the effects of individual dairy foods on cardiovascular events in male and female populations.

## Introduction

Cardiovascular disease (CVD) is the leading cause of death globally, with more people dying annually from CVD than from any other cause [1]. Coronary heart disease (CHD) is the most common type of heart disease, and it alone costs the United States an estimated $100 billion each year [2]. Diet has long been implicated in managing and reducing the risk of CVD [3]. A decade ago in America, it was suggested that consuming dairy foods (milk, cheese, and yogurt) in recommended amounts of three cup equivalents per day for persons 9 years and older could reduce the burden of CVD and its associated healthcare costs [4]. Since that time, observational data have indicated that the consumption of dairy foods is associated with better diet quality and nutrient status, and reduced risk of leading chronic diseases, including CVD and type 2 diabetes [5, 6].

CVD is an umbrella term that includes CHD and vascular diseases of the brain and blood vessels [7]. The etiology of CVD includes atherosclerosis, an inflammatory process that leads to hardening and narrowing of blood vessels, plaque build-up in vessel walls, and eventually plaque rupture, heart attack, or stroke [7]. Modifiable risk factors that play a role in the development of atherosclerosis include physical inactivity, overweight, and obesity [7].

Dairy foods such as milk, cheese, and yogurt are consumed by billions of people around the world [6]. Whole milk is primarily water (~88 %) and contains on average approximately 4 % fat, 3 % protein, 5 % carbohydrate (as lactose), and less than 1 % vitamins and minerals. Milk contributes significantly to intake of calcium, magnesium, riboflavin, and vitamin B_12_ globally [6].

The purpose of this review is to critically examine the recent literature, published between the years 2009 and 2013, on the relationship between dairy consumption and the risk of CVD. It focuses on the observational research of populations and their disease events and deaths.

Google Scholar (scholar.google.com) and PubMed (www.ncbi.nlm.nih.gov/pubmed) were searched for studies published between January 2009 and December 2013 that investigated the link between dairy consumption and CVD risk. Search terms used were “dairy,” “milk,” “cheese,” or “yogurt” in combination with “cardiovascular disease,” “coronary heart disease,” “atherosclerosis,” “heart,” or “stroke”. References of review articles were examined for additional relevant studies. This review included all observational studies that examined the association between dairy consumption and risk of CVD and adjusted for potential confounding variables. Excluded from the review were studies that pertained to dairy consumption and known risk factors for CVD, such as elevated blood lipids, hypertension, and type 2 diabetes. Recent reviews on the subject of dairy/dairy components and their relationship with cardiometabolic health [8], blood pressure [9], and type 2 diabetes [10] are available for reference.

## Review of Recent Observational Evidence

Eighteen observational studies met the inclusion criteria for review and are summarized in Table 1, including results from fully adjusted models. Whereas total dairy consumption does not appear to contribute to CVD risk or CVD death, cultured dairy foods, including yogurt and cheese, appear to be inversely related to CVD risk. When examined by fat content, however, the relationship between dairy foods and CVD yields inconsistent results. The variability in findings may be explained by the inclusion criteria for dairy foods and their macronutrient composition, how dairy foods are consumed (alone or as mixed dishes, such as pizza), and the potential for residual confounding [11].

**Table 1:** Epidemiological studies published between 2009 and 2013 that investigated the association between dairy consumption and risk of cardiovascular disease*

Reference	Region	Study / Objective	Population	Dietary Assessment	Adjustments	Time	Results	Risk
Bonthuis et al*.* (2010)[12]	Australia	Prospective study to determine if intake of dairy products or related nutrients is associated with mortality due to CVD, cancer and all causes	1,529 Australian men and women, 25–78 years of age	FFQ; Dairy = milk (low-fat, fat free) low-fat yogurt, cheese, whole milk, cream, ice cream, custard	Calcium intake, age, sex, energy intake, BMI, alcohol intake, education, PA, smoking, supplement use, medications, β-carotene treatment, diet, medical history	14.4 y BL: 1992, 1994, 1996	Highest intake of full-fat dairy (339 g/d) had reduced CVD death compared to lowest (34 g/d) intake (HR = 0.31; 95 % CI: 0.12–0.79, *P* = 0.04).	↓
							NS association between dairy intake and CVD, cancer or total mortality.	↔
Elwood et al*.* (2010)[19•]	Europe, North America, Asia, South Pacific	Meta-analysis of prospective studies to determine the association between dairy consumption and relative risk for vascular diseases	1,000,000+ multiethnic adult men and women from 38 published studies (broken down into different meta-analyses)	Collection method NR; Dairy = milk (whole, semi-skimmed, fat-free), cheese, butter, cream, yogurt, ice cream	Multiple adjustments Meta-analyses tested first for homogeneity of results Pooled RR from heterogenic studies were determined from weighted natural logs of each individual RR by the inverse of the variance	Variable, per analysis	Highest vs. lowest dairy consumption inversely related to all-cause deaths (RR = 0.87; 95 % CI: 0.77, 0.98); ischemic heart disease (RR = 0.92; 95 % CI: 0.80, 0.99); stroke (RR = 0.79; 95 % CI: 0.68, 0.91); and incident diabetes (RR = 0.85; 95 % CI: 0.75, 0.96).	↓
Warensjö et al. (2010)[28]	Sweden	Nested prospective case–control study to determine the association between dairy fat intake, assessed by serum 15:0 and 17:0 fatty acids and FFQ, and first MI	444 cases and 556 controls, Swedish men and women, 49–64 years of age	serum 15:0, 17:0 fatty acids, FFQ; Dairy = cream, cheese, fermented products, milk, ice cream	Matched for age, sex, date of examination, geographic region Adjusted for PA, BMI, smoking, diet, education, apo-ratio, SBP, diabetes prevalence	3–4 y BL: 1987–1999	NS association between dairy fat intake and first MI in fully adjusted model.	↔
Ferland et al. (2011)[26]	Canada	Cross-sectional study to determine the association between dairy product intake and body weight and CVD risk profile among a population undergoing a dietary transition	543 Nanavik Inuit (Canada) men and women, 18–74 years of age	FFQ; Dairy = milk, yogurt, ice cream, cheese and butter	Age, sex, smoking, education, dietary patterns	Data collected over 5 weeks	NS association between dairy consumption and CVD risk factors.	↔
Goldbohm et al. (2011)[18]	The Netherlands	Prospective study to determine the association between dairy product consumption and risk of death from all causes, IHD and stroke	120,852 Dutch men and women, 55–69 years of age	FFQ; Dairy = milk, yogurt, buttermilk, quark, and dishes in which these foods were used	Age, smoking, PA, education, BMI, energy, multivitamin use, MUFA intake (energy-adjusted), PUFA intake (energy-adjusted), vegetable intake, fruit intake, alcohol intake	10 y BL: 1986	Fermented full-fat milk inversely associated with all-cause mortality (RR = 0.91; 95 % CI: 0.86, 0.97 per 100 ml/d) in men and (RR = 0.92; 95 % CI: 0.85, 1.00) women.	↓
							Dairy fat or butter intake associated with all-cause and IHD mortality (per 10 g/dl rate ratio_MORTALITY_: 1.04; 95 % CI: 1.01, 1.06) in women only.	↑
Ivey et al. (2011)[32]	Australia	Prospective study to determine the relationship between consumption of milk, cheese and yogurt and CCA-IMT	1,080 Australian women, 70+ years of age	FFQ; Dairy = milk, cheese, yogurt	Age, BMI, energy intake, energy expended in PA, use of vascular medication, diabetes, history of vascular disease, smoking	3 y BL: 1998	>100 g/d of yogurt inversely associated with CCA-IMT (−0.023 mm; *P* = 0.003).	↓
							NS association between total dairy, milk, or cheese and CCA-IMT.	↔
Soedamah-Muthu et al. (2011)[16]	Europe North America Asia	Dose–response meta-analysis to determine the associations of milk, total dairy products, and full- and low-fat dairy intakes with the risk of CVD and total mortality	611,430 multiethnic men and women, 34–80 years of age, from 17 published studies	Questionnaires (10 validated FFQ out of 17 questionnaires); Dairy = milk, cheese, yogurt, butter, ice cream (varied by study) Meta-analysis studied only total dairy, total low-fat dairy, total high-fat dairy	Food Standards Agency used to convert servings or other units into g/d Multiple adjustments Meta-analyses tested first for homogeneity of results	14 y BL: 1960–2002	Inverse association between milk intake and risk of CVD (RR: 0.94 per 200 mL/d; 95 % CI; 0.89, 0.99).	↓
							NS association between milk and risk of CHD, stroke or total mortality.	↔
							NS association between whole-milk dairy and low-fat dairy per 200 g/d with CHD.	↔
Sonestedt et al*.* (2011)[20•]	Sweden	Prospective study to determine the association between milk, cheese, cream, and butter and incidence of CVD	26,445, Swedish men and women, 44–74 years of age	Diet history questionnaire; Dairy = milk, cheese, cream, butter	Age, season of data collection, energy intake, BMI, smoking habits, alcohol consumption, PA, education, anti-hypertensive therapy, lipid-lowering therapy, diet	12 y BL: 1991–1996	Highest vs. lowest intake of total dairy associated with 12 % decreased incidence of CVD (HR = 0.88; 95 % CI: 0.77, 1.02, *P* = 0.05).	↓
							NS association between milk intake (regardless of fat content) or butter and incidence of CVD	↔
							Highest vs. lowest intake of fermented milk associated with 12 % decreased incidence of CVD (HR = 0.88; 95%CI: 0.77, 1.00, *P* = 0.04).	↓
Aslibekyan et al. (2012)[27]	Costa Rica	Case–control study to determine the association between dairy intake, assessed by adipose 15:0 and 17:0 fatty acids and FFQ, and the risk of nonfatal MI	3,630 Costa Rican men and women, 48 – 74 years of age	Adipose 15:0 and 17:0 fatty acids, FFQ; Dairy = butter buttermilk, cheese, cream, ice cream, butter / margarine mix, milk (whole and semi-slimmed), and yogurt	Age, sex, area of residence, income, smoking status, total energy intake, PA, waist-to-hip ratio, alcohol intake, diet, adipose FA, self-reported history of hypertension, diabetes and hyper-cholesterolemia	Data collected over 10 y BL: 1994	Biomarker data: NS association between adipose 15:0 or 17:0 fatty acids and risk of MI.	↔
							FFQ data: NS association between dairy intake and risk of MI.	↔
Avalos et al. (2012)[38]	North America	Prospective study to determine the association between dairy consumption and CHD	751 Caucasian American community-swelling men and 1008 women, 50–93 years of age	FFQ; Dairy = milk (whole, fat-free) cream, ice cream, yogurt, cheese, low-fat cheese, cream cheese, cottage cheese, butter, hot chocolate, milk chocolate	Age, BMI, diabetes, hypertension, LDL-cholesterol, oestrogen use in women	16.2 y BL: 1984–1987	NS association between total dairy and risk of CHD	↔
							Higher intake of low-fat cheese and fat-free milk associated with increased risk of CHD in women only. HR = 1.48 (95 % CI 1.02, 2.16) for low-fat cheese and HR = 2.32 (95 % CI 1.57, 3.41) for fat-free milk.	↑
Dalmeijer et al. (2012)[25]	The Netherlands	Prospective study to determine the relationship between total dairy intake and dairy subtypes (whole-milk, low-fat, milk and milk products, cheese, and fermented dairy) with incident CHD and stroke	33,625 Dutch men and women, 21–64 years of age	FFQ; Dairy = all dairy food products except for butter and ice cream	Age, sex, total energy intake, smoking, BMI, education, PA, diet	13 y BL: 1993–1997	NS association between total dairy intake or dairy subtypes and risk of CHD or stroke.	↔
de Oliveira Otto et al. (2012)[29•]	North America	Prospective study to determine the association of saturated fat consumption from dairy and meat with the incidence of CVD events	5,209 multiethnic American men and women, 45–84 years of age	FFQ; Dairy = all dairy, including from mixed dishes	Sex, age, race-ethnicity, energy intake, study site, education, PA, alcohol, smoking, BMI, supplement use, cholesterol medication, diet	10 y BL: 2000	Saturated fat from dairy inversely associated with CVD risk HR = 0.79 (95 % CI: +5 g/d) and 0.62 (95 % CI: +5 % energy).	↓
							Substitution of 2 % of energy from meat saturated fat with dairy saturated fat = 25 % lower CVD risk HR = 0.75 (95%CI: 0.63, 0.91).	↓
							NS association between butter and CVD risk.	↔
							Saturate d fat inversely associated with CVD risk HR = 0.86 (95 % CI: +5 g/d) and 0.71 (95 % CI: +5 % energy).	↓
Larsson et al. (2012)[30]	Sweden	Prospective study to determine the association between consumption of total low-fat, full-fat and specific dairy foods and risk of stroke	74,961 Swedish men and women, 45–83 years of age	FFQ; Dairy = milk (whole, low-fat), sour milk/yogurt, cheese, cream / crème fraiche	Age, smoking, education, BMI, PA, aspirin use, history of hypertension, diabetes, family history of MI, total energy intake, alcohol intake, diet	10.2 y BL: 1997	Highest vs. lowest quintile of low-fat dairy consumption inversely related to risk for total stroke (RR = 0.88; 95%CI: 0.80, 0.97, *P* = 0.03) and cerebral infarction (RR = 0.87; 95 % CI: 0.78, 0.98, *P* = 0.03).	↓
							NS association between total dairy, full-fat dairy, milk, sour milk/yogurt, cheese, and cream / crème fraiche and stroke risk.	↔
Soedamah-Muthu et al. (2012)[15]	Europe	Prospective study to determine if intakes of total dairy, full-fat dairy, low-fat dairy, milk, and fermented dairy products were related to type 2 diabetes, CHD, and mortality	4,526, English men and women, mean 56 years of age	FFQ; Dairy = total dairy (all except butter and ice cream), full-fat dairy (full-fat cheese, yogurt, milk, pudding), low-fat dairy (cottage cheese, low-fat milk), total milk (whole, low-fat), low-fat milk (semi-skimmed, fat-free); fermented dairy (yogurt, cheese), yogurt, total cheese	Age, ethnicity, employment grade, smoking, alcohol intake, BMI, PA, family history of CHD or hypertension, diet	10 y BL: 1985 – 1988	NS association between total dairy and types of dairy with CHD or type 2 diabetes.	↔
							NS association between total dairy and all-cause mortality	↔
							Fermented dairy intake associated with 35 % decreased risk all-cause mortality (HR = 0.65; CI: 0.47, 0.90, *P* < 0.01)	↓
Kondo et al. (2013)[17]	Asia	Prospective study to determine the association between consumption of milk and dairy products and CVD death	9,243 Japanese men and women, 30+ years of age	FFQ; Dairy = 93 % milk and dairy product consumption was in the form of milk.	Age, BMI, alcohol intake, smoking, history of diabetes, use of anti-hypertensives, work category, total energy intake, systolic blood pressure, serum total cholesterol, diet	24 y BL: 1980	Each 100 g/d increase in consumption of milk and dairy products inversely associated with deaths from CVD, (HR = 0.86; 95 % CI: 0.74, 0.99, *P* = 0.05) in women only.	↓
Louie et al. (2013)[13]	South Pacific	Prospective study to determine the effects of habitual dairy consumption and the risk of 15–year CVD mortality	2,900 Australian men and women, 49–97 years of age	FFQ; Dairy = milk (whole, semi-skimmed, fat-free), cheese (whole milk, fat-free), dairy desserts	Age, sex, total energy intake, BMI, weight change, previous MI, previous stroke, smoking, hypertension, type 2 diabetes, hypertensive medication use, statin use, diet	15 y BL: 1992–1994	NS association between baseline consumption of dairy foods and risk of CHD, stroke and combined CVD mortality.	↔
Patterson et al. (2013)[21]	Sweden	Prospective study to determine the association between total and specific dairy food intakes and incidence of MI	33,636 Swedish women, 48–83 years of age	FFQ; Dairy = (full-fat, semi-skimmed, fat free), cultured milk / yogurt, cheese, cream / crème fraiche, butter (in cooking, on bread, both)	Diet, smoking, PA, waist-to-hip ratio, alcohol consumption, hypertension, high cholesterol, family history of MI, education, aspirin use, post-menopausal hormone therapy	11.6 y BL: 1997	Highest vs. lowest quintile of total dairy food intake associated with 27 % decreased incidence of MI (HR = 0.77; 95 % CI: 0.63, 0.95, *P* = 0.047).	↓
							Highest vs. lowest quintile of total cheese associated with 26 % decreased incidence of MI (HR = 0.74; 95 % CI: 0.60, 0.91, *P* = 0.006).	↓
							NS association between milk, cultured milk or cream, and MI (quartiles)	↔
							Highest vs. lowest quartile of full-fat cheese associated with 17 % decreased incidence of MI (HR = 0.83; 95 % CI: 0.68, 1.01, *P* = 0.035).	↓
van Aerde et al. (2013)[14]	The Netherlands	Prospective study to determine the relationship between type of dairy intake and CVD and all-cause mortality	1,956 Dutch men and women, 50–75 years of age	FFQ; Dairy = total dairy (all except butter and ice cream), full-fat dairy (full-fat cheese, yogurt, milk, pudding), low-fat dairy (cottage cheese, low-fat milk), total milk (whole, low-fat), low-fat milk (semi-skimmed, fat-free); fermented dairy (yogurt, cheese), yogurt, total cheese	Age, sex, BMI, smoking, education, total energy intake, alcohol consumption, PA, diet	12.4 y BL: 1989	Each standard deviation increase in full-fat dairy intake associated with 32 % increased incidence of CVD mortalit y (HR = 1.32; 95 % CI: 1.07, 1.61)	↑
							NS association between total dairy, low-fat dairy, milk and milk products, fermented dairy, or cheese, and incidence of CVD or all-cause mortality.	↔

*Abbreviations: BL, baseline; BMI, body mass index; CCA-IMT, common carotid artery intimamedia thickness; CHD, coronary heart disease; CVD, cardiovascular disease; FFQ, food frequency questionnaire; HR, hazard ratio; MI, myocardial infarction; NS, non-significant; PA, physical activity; RR, relative risk; SBP, systolic blood pressure

## Dairy and Risk of All-Cause or CVD-Specific Mortality

Four prospective studies that were reviewed observed no significant association between total dairy consumption and all-cause or CVD-death [12–15]. Correspondingly, a dose–response meta-analysis on the relationship of milk and total dairy product consumption with the risk of CVD observed no significant association between total dairy or milk consumption and risk of CHD, stroke, or all-cause mortality [16]. One prospective study of European adults observed a 35 % decreased incidence of all-cause mortality associated with cultured dairy intake (HR = 0.65; CI: 0.47, 0.90, *P* < 0.01) [15]. A prospective study of Japanese adults detected a 14 % decreased risk of CVD-specific mortality associated with each 100 g/day increase in milk consumption (HR = 0.86, 95 % CI: 0.74, 0.99), an observation made in women only [17]. Results from these investigations indicate that dairy consumption does not contribute to the risk of all-cause or CVD-specific mortality.

Observations reported in studies examining the relationship between fat content of dairy foods consumed and all-cause or CVD-specific mortality were inconsistent. In a prospective study of Dutch adults, an inverse association between fermented whole milk consumption and all-cause mortality was observed [18]. In that same study, however, dairy fat and butter intake, as calculated from the Dutch Food Composition Table, was associated with a 4 % increase in all-cause and CHD death in women (rate ratio_mortality_ per 10 g/d: 1.04; 95 % CI: 1.01, 1.06) [18]. These conflicting results may be attributable to the fact that the authors estimated dairy fat intake by combining all dairy foods reported – including as part of mixed dishes – and calculating the total, but reported the results regarding individual dairy foods based on information collected through food-frequency questionnaires (FFQ). In another prospective study of Dutch adults, each standard deviation increase in full-fat dairy intake (including milk, yogurt, curds, custards, and ice cream) as determined by FFQ was associated with 32 % higher risk of CVD mortality (95 % CI: 7–61 %) [14]. This was in contrast to the finding that total dairy, milk and milk products, fermented dairy, or cheese were not related to all-cause of CVD mortality in this same study, illustrating how difficult it can be to draw conclusions based upon categorizing intake. In this particular study, cases of CVD-mortality were limited. The population included older adults, 50–75 years of age, who were self-reporting their intake, and therefore, extrapolating these data to the broader population is not appropriate [14]. In a prospective study of Australian adults, however, it was observed that whereas there was no significant association between dairy intake and all-cause or CVD-death, the highest (339 g/day) versus the lowest (34 g/day) intake of full-fat dairy products (including whole milk, cream, ice cream, and custard) as determined by FFQ was associated with 69 % reduced incidence of CVD mortality (HR: 0.31; 95 % CI: 0.12–0.79, *P* = 0.04) [12]. Men and women ages 25–78 were observed in this study, and more potential confounders were included in adjusted models than in the other studies previously reviewed. The population, however, was smaller.

Taken together, the results from these studies support the need for research on specific dairy foods with well-defined macronutrient compositions in order to clarify our current understanding of the relationship between full-fat dairy consumption and all-cause or CVD-specific mortality.

## Dairy and Risk of CVD

A series of meta-analyses of prospective studies examined the relationships between dairy consumption, defined as milk (whole, semi-skimmed, fat-free), cheese, butter, cream, yogurt, and ice cream, and the relative risks for CVD, including CHD and stroke. One meta-analysis including nine prospective studies indicated that the highest versus lowest amounts of total dairy consumption reduced the relative risk of CHD by 8 % (RR = 0.92; 95 % CI: 0.80, 0.99, *P* < 0.05) [19•]. Another meta-analysis including 11 studies indicated that the highest versus lowest amounts of total dairy consumption reduced the risk of stroke by 21 % (RR = 0.79; 95 % CI: 0.68, 0.91) [19•]. Unfortunately, the amount of each specific dairy food in each of the studies included in the meta-analyses could not always be determined, making it difficult to draw firm conclusions as to whether one dairy food is more or less associated with risk of CHD or stroke than another. Furthermore, the definition of “highest” and “lowest” intake differed between studies. In one study, the “highest” intake was defined as “milk used on cereal.” In another it was defined as “more than two pints of milk per day,” and “lowest” intake was defined as “no milk consumed at all.” These inconsistencies make it nearly impossible to perform comparative analysis of risk reduction with any level of certainty. Nonetheless, testing for heterogeneity of results among studies yielded non-significant differences, thus rendering the meta-analyses by Elwood and colleagues [19•] some of the most comprehensive reviews on dairy consumption and CVD risk endpoints available in the literature today. Results of the meta-analyses demonstrate that dairy consumption is associated with a reduced risk of all-cause death and CVD, specifically ischemic heart disease and stroke [19•]. A dose–response meta-analysis of over 600,000 multiethnic adults observed an inverse association between milk intake and CVD risk, with a 6 % decreased risk associated with each 200 ml/day of milk consumed [16].In a prospective study of Swedish adults, total dairy consumption defined as milk, cheese, cream, and butter, collected from a diet history questionnaire, was associated with a 12 % decreased incidence of CVD (HR = 0.88; 95 % CI: 0.77, 1.02, *P* = 0.05), defined as incidence of fatal or non-fatal heart attack or stroke[20•]. When specific dairy foods were examined, this result was attributable to the consumption of fermented milk, in which the highest (~ 200–300 g/day) versus lowest (0 g/day) intake was associated with a 15 % reduced incidence of CVD (HR = 0.85, 95 % CI: 5 – 24 %, *P* = 0.03) [20•]. Neither milk (full-fat or low-fat) nor butter was associated with incidence of CVD in this study [20•]. The findings from this study are important for several reasons: they were obtained by utilizing a reliable method of determining food intake, they were reported from fully adjusted models, and they differentiated between dairy foods and their relationship with the incidence of CVD. A prospective study of Swedish adults observing women only demonstrated that the highest (8.4 servings per day) versus lowest (2.2 servings per day) quintile of total dairy food intake, defined as milk (all fat varieties), cultured milk/yogurt, cheese, cream/crème fraiche, and butter (in cooking, on bread, and both) was associated with a 23 % decreased incidence of myocardial infarction (MI), commonly referred to as heart attack (HR = 0.77, 95 % CIL 0.63, 0.95, *P* < 0.05) [21]. When specific dairy foods were examined, a 26 % decreased incidence of MI (HR = 0.74;–95 % CI: 0.60, 0.91, *P* = 0.006) was observed in the highest versus lowest quintile of cheese intake, with the highest versus lowest quartile of full-fat cheese associated with a 17 % decreased incidence of MI (HR = 0.83; 95 % CI: 0.68, 1.01, *P* = 0.035) [21]. In this study, there was no association between milk, cultured milk, or cream and MI [21]. In an attempt to better understand the relationship between total dairy/cheese consumption and incidence of MI, researchers further adjusted for calcium, phosphorus, magnesium, and potassium in these groups only. The inverse relationship between total dairy and MI was attenuated after adjustment for calcium and phosphorus, and the inverse relationship between cheese intake and MI was attenuated after adjustment for calcium, indicating a role for these dairy nutrients in the reduction of heart disease risk [21]. Researchers also investigated the relationship of butter in cooking versus used on bread, observing a 34 % increased incidence of MI in women who used butter on bread but not in cooking, a result not attenuated by adjustments for oil and margarine use in cooking [21]. The results of these studies indicate the complexity of dairy foods and the differences in CVD risk depending upon the type of dairy food consumed. Whereas total dairy and cheese reportedly had inverse relationships with CVD risk, butter (as a spread) was associated with disease but total butter consumption was not. The need for studies that differentiate between dairy foods and their relationship with risk of CHD and stroke is supported by these findings. One consistent outcome from the studies, however, was that total and cultured dairy, including milk, cheese, and yogurt, regardless of fat content, did not appear to contribute to the risk of CVD.

One commonly accepted nutrition strategy for reducing CVD risk is maintaining a diet low in saturated fat [22]. Although this strategy has recently been called into question [23, 24], as long as saturated fat reduction remains part of the heart-healthy dialogue, the association between the fat content of dairy products and risk of CVD will remain relevant. In the dose–response meta-analysis of over 600,000 multiethnic adults (discussed previously in this review), no significant association was observed between full-fat or low-fat dairy consumption (up to 200 mg/d) and risk of CHD [16]. A prospective study of Dutch adults yielded similar results, reporting no observed relationship between total dairy intake (or dairy subtypes, including all dairy foods except butter and ice cream), and risk of CHD or stroke [25]. In a very small cross-sectional study of Nunivak Inuit adults (Canada) there was no observed association between full-fat dairy foods, including milk, yogurt, ice cream, cheese, and butter, and risk factors for CVD [26]. Although the study was small (543 participants) and short in duration (5 weeks), it gave a glimpse into the relationship between diet and health in a population whose dietary patterns are in transition [26]. The results of these studies indicate that fat-containing dairy foods do not contribute to CVD risk despite their saturated fat content.

Some studies have used 15:0 and 17:0 fatty acids, which are exogenous fatty acids that serve as biomarkers of dairy fat intake, to examine the effect of full-fat dairy consumption on risk of MI. There was no association between full-fat dairy intake and MI among Costa Rican adults from a prospective case–control study that utilized adipose 15:0 and 17:0 fatty acids alongside FFQ to determine consumption of full-fat dairy (defined as butter, buttermilk, cheese, cream, ice cream, butter/margarine mix, milk [whole and semi-slimmed], and yogurt) among participants [27]. Similarly, no significant association between full-fat dairy intake and MI was detected in a prospective study of Swedish adults in which full-fat dairy consumption, (including cream, cheese, butter, fermented products, milk, and ice cream) was assessed by serum 15:0 and 17:0 fatty acids in combination with FFQ [27]. In this study, however, data from the serum fatty acid biomarkers indicated a 26 % reduced risk for first MI associated with full-fat dairy consumption in women [28]. When specific dairy foods were examined, the highest (> 219 g/day) versus lowest quartile (> 33 g/day) of fermented milk intake was associated with a reduced risk of first MI [28]. Furthermore, there was an interaction observed for cheese, with the highest (~20–24 g/day) versus lowest (~ < 7 g/day) quartile of reported intake associated with a reduced risk of first MI in men only [28]. The significance of these findings was attenuated in fully adjusted models, indicating a neutral association between full-fat dairy food intake and first MI in men and women. One study was designed to examine the association between the saturated fat consumed from dairy or meat with the incidence of CVD events in a multiethnic adult population. The data indicated that total saturated fat intake was associated with a reduced incidence of CVD and that saturated fat specifically from dairy foods was also inversely associated with CVD incidence, with a 21 % decrease in hazard ratio for every 5 g/day increase of dairy saturated fat (HR = 0.79, 95 % CI: +5 g/d) and a 38 % decrease in hazard ratio for every 5 % increase of daily energy from dairy saturated fat (HR = 0.62, 95 % CI: +5 % energy) [29•]. The researchers also observed a 25 % lower hazard ratio for CVD risk (HR = 0.75, 95 % CI: 0.63, 0.91) for each 2 % of energy substitution of dairy saturated fat for meat saturated fat, with no significant association between butter and CVD risk detected [29•]. This study illustrates a paradigm shift in how nutrition scientists think about saturated fat and heart disease. Not only does it bring into question the association between saturated fat and heart disease, but it points out that food sources of saturated fat differ, and to classify a complex food matrix by its fat content alone is too simplistic an approach to nutrition science. The results of the studies reviewed here implicate cultured dairy, including cheese, as having inverse relationships with CVD risk despite their saturated fat content. When assessed from fatty acid biomarkers and FFQ, the relationship between total butter intake and CVD in these investigations was neutral. These findings underscore the importance of examining the contribution of individual dairy foods to the risk of developing CVD.

It has been long accepted among the nutrition community that low-fat dairy foods do not contribute to the risk of CVD, and fit into a heart-healthy diet plan [5]. Accordingly, in a prospective study of Swedish adults, whereas there was no significant association between total dairy, full-fat dairy, milk, yogurt, cheese, or cream and risk of stroke, there was a 12 % reduced risk for total stroke (RR = 0.88, 95 % CI, 0.80, 0.97, *P* = 0.03) and a 13 % reduced risk for cerebral infarction (RR = 0.87, 95 % CI, 0.78, 0.98, *P* = 0.03) in the highest versus lowest quintile of low-fat dairy consumption, indicating a beneficial relationship between low-fat dairy intake and risk of stroke [30]. This was in contrast to the findings reported from a prospective study of Caucasian American adults in which it was observed that women who reported “nearly daily” low-fat cheese and fat-free milk consumption had an increased incidence of CHD compared to those who reported “rarely/never” consuming low-fat cheese and fat-free milk. This was a surprising observation in light of the fact that there was no significant association between total dairy intake and risk of CHD in this population [31]. The population was a small cohort of older, community-dwelling adults that reported intake through FFQ. Despite adjustments for multiple confounding variables, the small, unrepresentative population may have been a partial contributing factor, as well as failure by the researchers to adjust for other dietary components and patterns. Nonetheless, the results of these studies indicate that the association between specific dairy foods and CVD risk may differ depending upon total dietary pattern as well as the composition of the dairy food studied (i.e., whether it is cultured or not, contains fat, or some other aspect of the food matrix). Furthermore, differences in how males and females respond metabolically to diet may play a role in these relationships. Therefore, further research is needed pertaining to the effects of individual dairy foods on CVD risk in both male and female populations.

## Dairy and Atherosclerosis

One prospective study in women 70 years of age and older examined the relationship between consumption of milk, cheese, and yogurt, and common carotid artery-intima media thickness (CCA-IMT), a measure of atherosclerosis progression. Whereas there was no significant association detected between total dairy, milk, or cheese and CCA-IMT, consumption of greater than 100 g/day of yogurt, on average, was associated with lower CCA-IMT (*P* = 0.03) [32]. This study, again, points to an inverse relationship between cultured dairy and CVD risk, although further research is needed to better understand this relationship.

## Potential Mechanisms

The mechanisms by which dairy foods, regardless of fat content, may reduce the risk of CVD have not been fully elucidated. A recent review of dairy components and risk for cardiometabolic syndrome explores some of the proposed mechanisms by which dairy may beneficially affect CVD risk [8]. In brief, the milk fat component of dairy contains over 400 unique fatty acids, many of which are not found in other foods [33]. Milk fat contains saturated, monounsaturated, and polyunsaturated fatty acids of varying chain lengths and configurations. The study of milk fatty acid chemistry is complex, and some studies have suggested that bioactive fatty acids in milk fat are responsible for anti-inflammatory and improved metabolic effects [34, 35]. In addition, the minerals contained in dairy foods, such as calcium, magnesium, phosphorus, and potassium, have been implicated in the management of elevated blood pressure and cardiometabolic syndrome [8]. Furthermore, calcium from dairy has been implicated in fecal fat excretion and the maintenance of healthy blood lipids [36]. Dairy foods are complex, however, and more recent evidence indicates that the dairy food matrix may be just as important as its individual components [37]. There are likely multiple mechanisms by which dairy consumption may help reduce CVD risk. Based on the totality of the evidence, it is recommended that Americans 9 years and older consume 3 servings of milk/milk products, including milk, cheese, and yogurt, per day [5].

## Conclusion

The review of observational evidence indicates that the consumption of milk, cheese, and yogurt does not contribute to the development of CVD. Some findings indicate a possible inverse association between milk and cultured dairy, including cheese and yogurt, and CVD, and further research is needed to better understand these relationships. Study results regarding the relationship between full-fat dairy consumption and CVD are inconsistent. To date, however, the body of literature gives plausibility to the hypothesis that full-fat dairy foods, including milk, cheese and yogurt, do not contribute to cardiovascular disease risk, and indeed may be inversely associated with it. Based on current evidence, additional prospective studies to determine if total dairy consumption is associated with CVD are neither prudent nor warranted at this time. Dairy fat is not eaten in isolation, and thus it must be studied in the context in which it is delivered. Future research time and investment should be focused on observations and interventions that will help elucidate the effect of individual dairy foods on CVD incidence and risk.

## Abbreviations

BL: baseline
CHD: coronary heart disease
CVD: cardiovascular disease
FFQ: food frequency questionnaire
HR: hazard ratio
MI: myocardial infarction
NS: non-significant
RR: relative-risk
